# Polysaccharide-Based Nanomaterials for Ocular Drug Delivery: A Perspective

**DOI:** 10.3389/fbioe.2020.601246

**Published:** 2020-12-10

**Authors:** Haozhe Yu, Wenyu Wu, Xiang Lin, Yun Feng

**Affiliations:** ^1^Institute of Medical Technology, Peking University Health Science Center, Beijing, China; ^2^Department of Ophthalmology, Peking University Third Hospital, Beijing, China; ^3^School of Chinese Medicine, The University of Hong Kong, Hong Kong, China

**Keywords:** polysaccharide-based nanomaterials, polysaccharides, ocular drug delivery, nanocarriers, nano

## Abstract

Ocular drug delivery is one of the most challenging issues in ophthalmology because of the complex physiological structure of the eye. Polysaccharide-based nanomaterials have been extensively investigated in recent years as ideal carriers for enhancing the bioavailability of drugs in the ocular system because of their biocompatibility and drug solubilization. From this perspective, we discuss the structural instability of polysaccharides and its impact on the synthesis process; examine the potential for developing bioactive polysaccharide-based ocular drug nanocarriers; propose four strategies for designing novel drug delivery nanomaterials; and suggest reviewing the behavior of nanomaterials in ocular tissues.

## Introduction

Given the emerging popularity of electronic devices and the corresponding changes in lifestyle, the incidence of ophthalmologic diseases has increased rapidly. It has been speculated that the number of blind individuals will add up to 702 million by 2050 (Flaxman et al., 2017). However, the efficacy of ocular drug delivery has remained challenging in clinical management, mainly because of the unique anatomy and complex physiological barriers of the eye. For anterior diseases, including dry eye syndrome, conjunctivitis, and keratitis, eye drops are recognized as common treatments because of their high accessibility at the ocular surface (Jian et al., 2017; Li et al., 2019). However, ocular surface barriers and associated stress responses, such as tearing, cause eye drops to have poor bioavailability, and hence frequent instilling is required to maintain an effective drug concentration (Bennett et al., 2020). The posterior of the eye mainly includes the vitreous body, choroid, and retina, which maintain distance from the ocular surface and barrier layers, such as the choroidal circulation region and the blood–retinal barrier. For the posterior, intravitreal injection has been adopted for direct drug delivery. Nonetheless, this method remains difficult for patients to accept due to possible side effects, such as endophthalmitis and trypanophobia and the associated economic burdens (Müller et al., 2017). The ideal ophthalmic drug delivery system should be simple and non-invasive with ensured bioavailability. In recent decades, increasing studies have reported nanoscale materials as novel ocular drug carriers considering their enhanced tissue permeation and sustained release characteristics (Lakhani et al., 2018; Lynch et al., 2019; Qamar et al., 2019).

**FIGURE 1:**
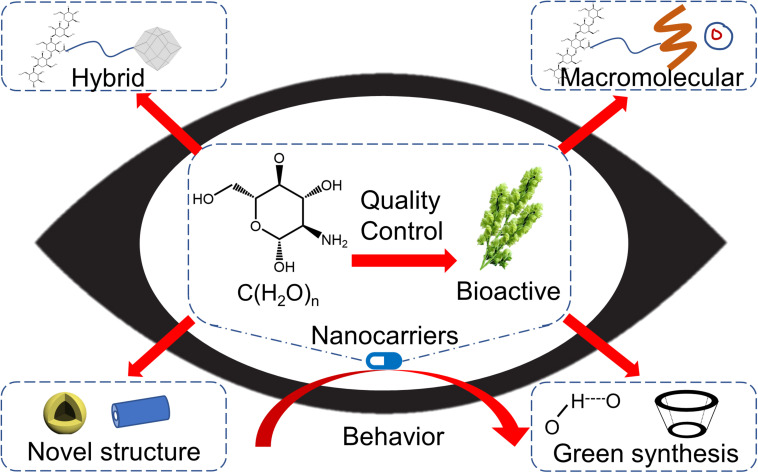
Future developing routes of polysaccharide-based nanomaterials for ocular drug delivery.

Polysaccharides are polymer compounds consisting of many monosaccharide molecules connected by glycosidic bonds. They can be divided into homopolysaccharides (composed of single monosaccharides, such as starch and cellulose) and heteropolysaccharides (composed of different monosaccharides, i.e., mucopolysaccharide). The physical characteristics of polysaccharides suggest distinct structural and physicochemical features, including chemical composition, degree of polymerization, number of branches, and surface charge, which are advantageous for designing drug carrier systems with adjustable characteristics in terms of drug loading content, release capacity, and biodistribution (Bernd et al., 2004; Furlani et al., 2019; Jin et al., 2020; Soliman et al., 2020). Compared with synthetic nanocarriers, polysaccharide-based nanomaterials exhibit a better performance in terms of drug retention and ocular permeability *via* the interpenetration of mucin chains, both directly and indirectly (Servais et al., 2018; George and Suchithra, 2019; Pathak, 2019). For example, chitosan, a type of polysaccharide with a linear structure and positive charge, can closely integrate with the cornea and conjunctiva, which have a negative surface charge. Consequently, this electrostatic interaction prolongs the retention time and enhances the penetration capability (Irimia et al., 2018). Moreover, polysaccharides are commonly present in the eye, such as hyaluronic acid, one of the major components of the vitreous body. Emerging studies have suggested the biocompatibility of polysaccharides and their derivative nanomaterials for ocular delivery (Nishikawa and Tamai, 1996; Nakagawa et al., 1997; De Oliveira Fulgêncio et al., 2012; Lodhi et al., 2020). Increasing evidence indicates their biosafety, good tolerance, and superior bioavailability, and polysaccharide-based nanocarriers have received considerable attention for clinical practice (Dubashynskaya N. et al., 2020). This perspective focuses on the structural instability of polysaccharides in synthesizing nanocarriers and provides a glimpse into their translational potential, including bioactive polysaccharide-based nanomaterials, novel strategies in nanocarrier design, and their behavior in ocular therapy (Figure 1).

## Structural Instability of Polysaccharides

Quality control is a critical challenge for translational studies of polysaccharide-based nanocarriers from bench to bedside. Control of the physical-chemical properties in large-scale nanocarrier synthesis has been fully discussed elsewhere (Nagarwal et al., 2010; Bazile, 2014; Felice et al., 2014). However, the structural instability of the parent polysaccharides is often overlooked. Polysaccharides are widespread in various natural sources, including animals, plants, microbes, and algae, and techniques for their isolation and purification are the foundation for developing polysaccharide-based nanomaterials (Mozammil Hasnain et al., 2019). Thus, polysaccharides are readily accessible compared with proteins and nucleic acids because the latter two can encounter conformational changes upon extraction. Nonetheless, emerging evidence suggests a sort of structural instability in polysaccharides during extraction. For example, an increase in the molecular weight of hemicellulose was observed in a high-temperature aqueous alkali environment. Compared with barium hydroxide, hemicellulose is more vulnerable to degradation when exposed to organic alkaline compounds (Bian et al., 2010, 2012). Moreover, the viscosity of chitosan extracted from fungi is lower than that of crustaceous chitosan (Żukiewicz-Sobczak et al., 2015). Recent studies have demonstrated that different sources and separation methods may result in variances in the purity, molecular weight, and substituents of hyaluronic acid and chondroitin sulfate, which subsequently affect their bioactivities (Abdallah et al., 2020). Chang et al. reported that chitosan extracted from *Auricularia* sp. elicited better antibacterial activities than commercial chitosan (Chang et al., 2019). These differences will most likely lead to unpredictable drug-release behaviors from the synthesized polysaccharide-based nanomaterials and thus cause unstable therapeutic effects *via* the ocular nanocarriers. Even for several commercialized polysaccharides, such as cellulose, chitosan, and hyaluronic acid, many structural properties, such as chain length, degree of polymerization, and polydispersity, can vary between batches, resulting in unreproducible experimental results. To deal with this instability, reports on nanomaterial preparation should include detailed structural characterization of the employed polysaccharides, whether extracted or commercialized. In this regard, given the functional importance of the physicochemical and biological properties of synthesized ocular nanocarriers, systematic review methods could promote classification algorithms for polysaccharide processes.

To scale up the production of polysaccharide-based nanocarriers, the structural instability must be overcome, particularly for polysaccharides with branched-chain or special functional groups like mucopolysaccharides. From this point of view, it seems difficult to standardize the industrial production of polysaccharides by pharmaceutical companies worldwide as well as specified polysaccharide derivatives without standard guidelines. Therefore, consensus and standards covering properties including chain length, polymolecularity, degree of substitution, and monosaccharide composition are a high priority.

## Bioactive Polysaccharide-Based Nanocarriers for Ocular Delivery

Existing studies on ocular nanocarriers have mainly focused on commercialized polysaccharides, including cellulose, chitosan, and hyaluronic acid, because of their their easy access and ready usage for nanocarrier synthesis without needing additional preparation. In contrast, little is known about the properties, modification reactions, and ophthalmological applications of polysaccharides extracted from natural sources. Many studies have suggested that certain natural polysaccharides, particularly those from plants, possess unique biological activities for the ocular system. *Lycium barbarum* polysaccharides have been reported to ameliorate dry eye disease, prevent oxidative damage in human trabecular meshwork cells, and preserve the function, ganglion cells, and pigment epithelium cells of the retina (Yang et al., 2017; Yang M. et al., 2020; Chien et al., 2018; Lakshmanan et al., 2019; Liu and Zhang, 2019; Liu L. et al., 2020). Carboxymethyl *Terminalia catappa* polysaccharides show therapeutic effects in the cornea from blue light-emitting diodes. Moreover, *Polygonatum sibiricum* polysaccharides could attenuate the damage from diabetic retinopathy, while dry eyes can be relieved by mucoadhesive *Bletilla striata* polysaccharide-based artificial tears (Chandel et al., 2019; Wang et al., 2019c; Thacker et al., 2020). Evidence has also demonstrated that phyto-based polysaccharides can be utilized for drug delivery systems upon graft modification or synergistic interaction *via* non-covalent stabilization with other commercial polysaccharides such as hyaluronic acid (Uccello-Barretta et al., 2013; Wang et al., 2019b). There is no doubt that leveraging this feature to develop dual-functional polysaccharide-based nanocarriers serving both carrier and therapeutic functions holds great potential from a clinical point of view.

The quality control of natural active polysaccharides has been challenging for developing ophthalmic drug-delivery systems and FDA approval. In addition to separation methods, it is also difficult to define certain polysaccharides with differential constituents, such as those from *P. sibiricum*, *P. cyrtonema*, *P. kingianum*, and *P. odoratum*; despite all being referred to as the so-called *P. sibiricum* polysaccharide, they present various saccharide mappings in high-performance gel permeation chromatography (Zhao et al., 2020). Moreover, emerging quality control methods for polysaccharides have been proposed, including polysaccharide receptor theory and multiple-fingerprint analysis (Cao et al., 2019; Li et al., 2020; Wang et al., 2020). In this context, the collaboration of experts in the fields of phytochemistry, analytical chemistry, and pharmacology is the highest priority to classify the structures of botanical polysaccharides for medical use.

## Typical Ocular Polysaccharide-Based Nanocarriers and Novel Design Strategies

Polysaccharides show high chemical activities because of their abundant functional groups, such as amino and hydroxyl groups (Yang Y. et al., 2020). Several reviews have summarized the synthetic protocols for common polysaccharide derivatives (Tiwari and Bahadur, 2019; Anpilova et al., 2020; Yang Y. et al., 2020). In the nanomaterial synthesis process, polysaccharides can be reacted under drastic conditions, such as high temperatures, salt contents, and shear forces, with relatively stable structures and biological activities compared with nucleic acids and proteins (Yuan et al., 2018; Garcia-Vaquero et al., 2019; Wang et al., 2019a; Chao et al., 2020; Chen B. et al., 2020). Therefore, by using polysaccharides and their derivatives, nanomaterials with different types of geometric structures can be prepared, from zero- to three-dimensional, through various synthetic methods, such as hydrothermal synthesis and co-precipitation (Ferreira et al., 2020; Alizadeh-Sani et al., 2021; Pan et al., 2021). Nanoparticles, nanocapsules, and nanomicelles are typical zero-dimensional drug carriers; nanoparticles are characterized by a uniform distribution of the drug and carrier, while the drug in the latter two is encapsulated in the carrier core. These nanomaterials improve the drug solubility and enhance the corneal penetration with prolonged retention times (Liu et al., 2019; Karava et al., 2020). One-dimensional polysaccharide-based nanomaterials are often used as excipients for their plasticity and low-encapsulation efficiency. For example, nanocellulose with its high mechanical strength can act as a reinforcing material (Taheri et al., 2020). Starting from one-dimensional nanofibers, the common two-dimensional fibrous membrane can be synthesized by electrospinning. Because of the high drug-loading capacity, long retention time, and sustained release of such membranes, they are often utilized as ocular inserts (Zheng et al., 2018). The properties of nanofibers dramatically affect the drug delivery performance (Mirzaeei et al., 2018; Yellanki et al., 2019). Nanogels are three-dimensional hydrophilic networks on the nanometer scale that are capable of encapsulating drugs or biological macromolecules while maintaining their native conformation. One important feature is that certain nanogels with functional groups (e.g., disulfide moieties) are stimuli-responsive. Exogenous stimuli, including temperature, pH, and ionic strength, can significantly influence the drug release kinetics and degradation rate; thus, nanogels are also called intelligent delivery systems (Chen S. et al., 2020; Lai et al., 2020).

Several strategies have been developed to maximize the drug delivery capacity of polysaccharide-based nanomaterials. A feasible design could be achieved using hybrid nanomaterials with polysaccharides. Polysaccharides can serve as stabilizers for other components, such as functional nanomaterials and biological macromolecules, in constructing polysaccharide-based nanohybrids (Zhan et al., 2019; Hammi et al., 2020). The participation of polysaccharides can also enhance the biocompatibility and biodegradability of exogenous materials, especially inorganic nanoparticles (Fan et al., 2019; Alaei et al., 2020; Dincă et al., 2020; Zheng et al., 2020). Santana et al. prepared ZnS-coated CdS quantum dots and ZnS-coated AgInS_2_ quantum dots by an aqueous route using chitosan conjugated with bevacizumab. Animal experiments showed no significant changes in electroretinography, intraocular pressure, histological, morphometric, or immunohistochemical examinations, indicating the good biocompatibility and biosafety of chitosan for ocular delivery (Santana et al., 2020). In addition, many studies have reported that the addition of precious-metal nanoparticles, such as silver, and carbon materials, such as graphene, can improve the antibacterial activity and release capacity (Shi et al., 2016; Joz Majidi et al., 2019; Asghar et al., 2020; Shah et al., 2020; Sun et al., 2020).

On the basis of small-molecule drugs, macromolecular drugs and cell therapy (e.g., stem cells) have attracted much attention because of their multi-target effects and promising clinical results, particularly for complicated ophthalmic diseases. Previous research has shown that hyaluronan–chitosan nanoparticles can be absorbed by the corneal and conjunctival epithelial cells and further assimilated, suggesting the potential of polysaccharide-based nanocarriers for gene therapy (de La Fuente et al., 2008a,b). Chaharband et al. synthesized chitosan–hyaluronic acid nanopolyplexes loaded with siRNA through an ionic gelation method, which could penetrate the vitreous and retina barriers. Intravitreal injection experiments demonstrated that the nanopolyplexes could reach the posterior of rabbits and effectively reduce the size of laser-induced choroidal neovascularization (Chaharband et al., 2020). Biological macromolecules such as peptides can also be used to form polysaccharide-based hybrid nanocarriers (Qian et al., 2019; Lu et al., 2020). Silva et al. developed chitosan–hyaluronic acid nanoparticles loaded with erythropoietin. *In vitro* permeation experiments showed rapid penetration into porcine conjunctiva followed by the sclera and cornea, with no cellular toxicity (Silva et al., 2020). Considering the therapeutic potential of stem cells for ophthalmological diseases, the development of polysaccharide-based cell-loading nanocarriers for further exploration is encouraging, especially for retinal and corneal stem cells.

Novel structural types of polysaccharide-based nanomaterials such as core-shell, hollow, and multi-layer structures have shown excellent drug delivery properties in terms of stability, drug-loading capacity, sustained release, corneal permeability, multi-stimuli sensitivity, and ocular bioavailability (Liu et al., 2018; Nie et al., 2019; Tan et al., 2019; Wei et al., 2020). Machado et al. synthesized a brimonidine-containing polymer-β-cyclodextrin membrane with graphene oxide nanosheets and poly-β-aminoester intercalation. The drug release experiment suggested an association between the drug release kinetics and the number of graphene oxide nanosheet layers, making it easy to implement time-controlled drug release in the ocular system (Machado et al., 2019). Luo et al. prepared dual-functional nanoparticles by modifying chitosan and ZM241385 onto the surfaces of hollow ceria nanoparticles loaded with pilocarpine. The hollow structure significantly improved the drug retention. In addition, chitosan and ZM241385 were able to penetrate the cornea, while ceria elicited antioxidant and anti-inflammatory functions. These nanoparticles exhibited a 42-fold longer period of lowering the intraocular pressure compared with that of commercial eye drops (Luo et al., 2020). Jiang et al. prepared a core-shell structured polysaccharide-based nanocarrier with a polycaprolactone shell and a chitosan core through a two-step emulsion method. The chitosan core was filled with bevacizumab *via* electrostatic interactions. These core-shell particles had a significantly improved capacity for long-term release of up to 3 months and possess good prospects for anti-VEGF therapeutics in clinical practice (Jiang et al., 2020).

In the synthesis of polysaccharide-based nanomaterials, some reagents may cause unpredictable toxicity toward normal tissues because of the limited available toxicological information compared with numerous synthetic compounds. Therefore, synthetic approaches based on green chemistry, such as self-assembly *via* hydrogen bonding, hydrophilic/hydrophobic interactions, Van der Waals forces, and electrostatic interactions, have attracted increasing interest (Han et al., 2020; Jin et al., 2020). Alqurshi et al. synthesized nanoparticles for delivering prednisolone acetate based on chitosan and sodium deoxycholate as a counterion through self-assembly. These nanocarriers performed better in terms of drug release behavior and anti-inflammatory effects in the guinea pig model compared with micronized gel (Alqurshi et al., 2019). Several studies have attempted to modify polysaccharides simply to obtain various non-covalent interactions (Dubashynskaya N. V. et al., 2020; Liu C.H. et al., 2020).

## Behavior of Polysaccharide-Based Nanomaterials in Ocular Tissue

Before further clinical trials in patients with polysaccharide-based nanomaterials, their biological behavior and potential influence on the ocular system must be determined. Although emerging studies have emphasized the biocompatibility and biosafety of polysaccharides, a few have reported concerns regarding worsening the ocular pathology, including slightly elevated inflammatory factors and decreased densities of cones and rods (Yang et al., 2008; Jiang et al., 2018). In particular, as the size of materials decreases to the nanoscale, whether the corresponding changes in physical and chemical properties will have toxic effects should be further studied based on structural nanotoxicology (Zielińska et al., 2020). There have been several studies focusing on the biosafety of polysaccharide-based nanocarriers for ocular compartments; however, data on long-term observations and drug formulation are still lacking (De Campos et al., 2004; De Salamanca et al., 2006; Prow et al., 2008; Lai et al., 2010; Zorzi et al., 2011; Lai, 2012; Ogunjimi et al., 2017). Furthermore, the biodegradation of polysaccharide-based nanocarriers in the ocular system remains far from satisfactory, despite the fact that polysaccharides easily degrade *in vivo* (Etienne et al., 2005; Nguyen et al., 2019). Lai et al. detected polysaccharide degradation from drug carriers in lysozyme-containing buffers, a key metabolic regulator also present in the aqueous humor of the eyes, and suggested the possible enzymatic degradation of polysaccharide-based nanocarriers post-injection (Lai and Luo, 2017). However, at the nanoscale, some specific questions remain unclear, including where and how the polysaccharide-based nanocarrier is decomposed, metabolized, and excreted; what metabolites are produced by their degradation; and the possible impacts on the physiological function of the eye, particularly for hybrid nanomaterials. Emerging evidence has demonstrated the biocompatibility and biosafety of polysaccharide-based nanocarriers based on material design, but there is a lack of effectiveness in real scenarios. In terms of the effect of controlled drug release in time-and-space evaluation, certain studies on polysaccharide-based nanocarriers, especially in the context of the physiological environment, remain controversial due to their differing animal models and research methodologies. In addition, as a sensory organ, visual function significantly affects patient compliance. Therefore, the influence of drug-delivery systems on visual quality should be considered. Nanoparticles are prone to aggregating because of their low surface energy, and as the size of aggregates increases, optometric diseases will emerge, such as vitreous opacity (Sultana et al., 2020). It is undeniable that the evaluation of visual quality is difficult, considering the physiological differences between human and animal models. Consequently, multi-disciplinary cooperation including ophthalmology, optometry, chemistry, materials science, and zoology should be encouraged. For instance, the development of biomimetic eye models based on engineering perspectives is expected to improve our understanding of the biological behavior of drug carriers (Bennet et al., 2020; Peng et al., 2020).

## Data Availability Statement

The original contributions presented in the study are included in the article/supplementary material, further inquiries can be directed to the corresponding author/s.

## Author Contributions

YF and HY devised the conception of the study. HY, WW, and XL wrote the manuscript. All authors contributed to the article and approved the submitted version.

## Conflict of Interest

The authors declare that the research was conducted in the absence of any commercial or financial relationships that could be construed as a potential conflict of interest.
